# Impact of Classical Counterconditioning (Quiet Kennel Exercise) on Barking in Kenneled Dogs—A Pilot Study

**DOI:** 10.3390/ani12020171

**Published:** 2022-01-11

**Authors:** Samantha Zurlinden, Stephany Spano, Emily Griffith, Sara Bennett

**Affiliations:** 1 College of Veterinary Medicine, North Carolina State University, 1060 William Moore Drive, Raleigh, NC 27607, USA; sbzurlin@ncsu.edu (S.Z.); srspano@ncsu.edu (S.S.); 2 Department of Statistics, North Carolina State University, 2311 Stinson Drive, Raleigh, NC 27607, USA; eghohmei@ncsu.edu

**Keywords:** barking, dogs, shelter, kennel, welfare, classical conditioning

## Abstract

**Simple Summary:**

Barking is a major source of noise pollution in dog kennels and negatively impacts the welfare of anyone within earshot, especially the dogs in the kennels themselves. It is crucial to have solutions to help reduce barking quickly and humanely that are easy to understand and put into place that also do not require a lot of resources such as time, expertise, or money. This study looked at the use of an exercise (Quiet Kennel Exercise—QKE) that uses classical counterconditioning (Pavlov) to help change the way the dogs feel when a person enters the kennel area from unpleasant to pleasant. This should help to reduce barking, especially that which is caused by negative emotions such as fear and frustration. After the initial baseline period of 5 days, people passing through the kennel tossed treats to the dogs no matter what the dogs did for 10 days. Data was collected three times a day for the entire study period. Sound level readings (decibels), number of dogs present, and number of dogs barking were recorded. Results showed improvement in the loudest volume recorded after the exercise was in place, fewer dogs barking over time, and each dog barking less each time. The most improvement was noticed in the afternoon.

**Abstract:**

Excessive barking is a major source of noise pollution in dog kennels and negatively impacts welfare. Because resources are often limited, minimizing barking in the simplest and most easily implementable way is imperative. This pilot study implemented a Quiet Kennel Exercise (QKE) that utilized classical counterconditioning to change the dogs’ negative emotional state (which can lead to barking) to a more positive emotional state. Therefore, barking motivation is reduced, so barking should decrease. This study aims to show proof of concept that decreasing barking through classical counterconditioning is effective. It was conducted in one ward of day-time boarding kennels at North Carolina State University College of Veterinary Medicine. Data was collected three times per day and included decibel readings, number of dogs present, and number of dogs barking during a 5-day initial baseline and 10-day intervention period. During baseline, people passing through the ward acted as they normally would. During intervention, passersby were asked to simply toss each dog a treat regardless of the dogs’ behaviors in the kennel. Descriptive results show improvement in maximum level of barking after QKE, fewer dogs barking over time, dogs barking less each time, and the most improvement noted in the afternoon.

## 1. Introduction

Excessive barking in facilities that house kenneled dogs is a recognized welfare issue [1,2,3,4]. Barking is a concern for dogs kenneled for any reason including, but not limited to, boarding facilities, working dog housing, laboratory units, veterinary clinics, and animal shelters. The majority of noise pollution in animal shelters is caused by barking from dogs that are housed there [3]. Dogs have a more sensitive sense of hearing than people, with the ability to hear sounds up to 4 times quieter than people [3]. People cannot hear sound frequencies above 20 kHz, but dogs can detect sound frequencies from 40 Hz to 50 kHz [2]. Dogs are most sensitive to sounds at frequencies from 500 Hz to 16 kHz, and their threshold of sensitivity is 24 dB lower than that of a person, which means sound damaging to people is likely to have an equal, if not more damaging effect on dogs [3]. The Occupational Safety and Health Administration (OSHA) mandates a hearing protection program for people when exposed to noise levels averaging at or above 85 dB for 8 h due to the risk of hearing damage caused by auditory neuronal cell death as a consequence of high noise level exposure [5]. The volume of noise in animal shelters and veterinary clinic kennels routinely measures greater than 100 dB, can be sustained at 95 dB for 15 min while people are present, and a single bark can reach this volume on its own [2,3,4]. Despite this information, there is no regulatory oversight for acoustic safety or noise mitigation for the dogs themselves housed in kennel environments. Scheifele et al. (2012) [4] performed Acoustic Brainstem Response (ABR) testing, also known as Brainstem Auditory Evoked Response (BAER) testing, in dogs housed in kennels for a six-month time period. Over half of the dogs evaluated experienced a greater than a 20 dB reduction in their hearing after being exposed to the kennel environment for those six months. In people, a change of greater than 10 dB indicates an important and concerning change in hearing [4], so the 20 dB change in dog hearing is an important welfare concern for dogs housed in kennel settings. Beerda et al. (1997) [6] exposed dogs to various volumes of sound up to 95 dB and monitored behavioral and physiological responses to these stimuli. They found the dogs displayed an increase in paw lifting, lowered body postures, body shaking, and snout licks, all of which were indicative of increased stress or a negative emotional state. Behavior changes were more pronounced with the increased decibel of sound exposure. Physiologically, one dog exposed to 95 dB had a clear response magnitude change in salivary cortisol level from before, during and after recovery from the noise. An increased heart rate up to 54% from baseline was also reported for this dog [6].

Additionally, chronic stress can be caused by excessive barking [7]. Chronic stress can impair immune function and consequently increase disease susceptibility [8]. Furthermore, the volume of sound in kennel environments has the potential to negatively impact the hearing and mental well-being of kennel workers and other animal species within hearing distance [2,9,10], particularly cats, small mammals and avian species that are considered a prey species for canids. Dogs who experience stress and anxiety in shelters and kennel environments are also suspected to be more susceptible to infections such as Chronic Infectious Respiratory Disease Complex (Kennel Cough, CIRDC). Skandakumar et al. (1995) [11] found that secretory IgA levels in dogs are decreased when dogs experience stress. Bey et al. (1981) [12] found that increased levels of mucosal IgA was one important factor associated in dogs that were able to confer resistance to clinical CIRDC. The data from these two studies present further argument that perhaps physical health is negatively impacted by emotional distress, and it is prudent to consider measures which could be taken to minimize stress in order to help improve physical and emotional welfare in kenneled dogs. The Quiet Kennel Exercise (QKE) is one humane and practical strategy that can be used to help reduce barking when people are present, although ideally it can be utilized in conjunction with other methods to help reduce barking when people are not present for maximal welfare improvement. Other studies have shown specific auditory stimulants [13,14] such as classical music and some audiobooks and olfactory stimulants [15] such as lavender and chamomile, may help encourage quiet resting behaviors in kenneled dogs, which indicates improved welfare as well as serving as a source of enrichment. Additionally, limiting visual contact between dogs by using even partial visual barriers can be useful for reducing excess vocalization and improving welfare [16].

It is important to also consider the volume of barking that is audible to other species housed in a shelter, and how their welfare is impacted. Tanaka et al. (2012) [17] found that housing cats in areas where barking is audible is likely to cause fear and stress for cats, which can lead to an increased incidence of upper respiratory infections and weight loss during shelter stays [17]. McCobb et al. (2005) [18] found that exposure to dogs and dog vocalization was the largest factor that negatively affected cats’ stress levels in different types of shelter housing [18]. Gourkow et al. (2014) [19] found that cats who perceive being threatened and/or show signs of stress and anxiety in shelter environments experience reduced levels of mucosal IgA concentrations, making them more susceptible to upper respiratory infections [19]. This necessitates medical treatment, which can create more stress and a longer length of stay (LOS) (the amount of time in the shelter, from intake to exit). The Association of Shelter Veterinarians (ASV)’s Guidelines for Standards of Care in Animal Shelters [20] clearly outlines the importance of behavioral modification in addition to architectural noise mitigation strategies in order to decrease the volume of sound in shelters.

Several studies have demonstrated that barking can negatively impact people who are involved with kenneled dogs in different capacities. Behavior is an important factor when people are making adoption decisions [21], and the average potential adopter only spends about 70 s in front of each kennel [22]. Additionally, if the ward or room where available dogs are housed is so loud as to be distressing or even painful for a person to walk into, potential adopters may be turned off from even looking at the adoptable dogs [2], especially if access to online first impressions are limited. This necessitates the importance of barking reduction in shelter dogs in order to make a good impression on potential adopters, allow the pet selection experience to be a more pleasant one, and hopefully reduce the dogs’ lengths of stay.

People who work and volunteer in animal shelters also are negatively impacted by the noise pollution ubiquitous in the shelter environment. This negative experience can feed into the already high risk of compassion fatigue and burnout. Compassion fatigue is defined as “the combined effect of secondary traumatic stress and cumulative burnout, a state of mental and physical exhaustion which is characterized by the loss of ability to nurture” [23]. Many people work or volunteer in shelters because they want to make a difference in the animal’s wellbeing [23]. By lowering the volume of sound caused by barking, the wellbeing of animals will be improved by human intervention. Having an intervention available that these people can actively provide empowers those very people at risk of developing compassion fatigue by giving them a way to create a direct beneficial impact on the animals in their care. Additionally, by lowering the volume of sound, the humans themselves are not experiencing as many negative stressors from the environment either and preserves their sense of hearing.

In kennels, dogs may bark due to unpredictable high levels of noise, novelty, lack of control of their environment, and disrupted routines [24], in addition to territorial communication and excitement [3,10,25]. Repeated exposure without the option to resolve the motivation often causes a negative emotional state due to fear, frustration, or anxiety [7]. The volume of noise in a kennel setting can increase dramatically following a disturbance, such as the presence of a visitor, or in anticipation of events [3]. It is important to keep in mind the goal of barking is considered one of communication, with many experts considering the main target to be people, along with other dogs [26]. The underlying motivations secondary to these signals not resulting in the expected communicative consequence is often frustration from those dogs anticipating the opportunity for social interaction that is then often thwarted, or due to agonistic (distance increasing) motivation such as fear or territorial behaviors in response to the presence of people or other unfamiliar dogs. In addition to anticipating social interactions, regardless of whether the dog is looking forward to the event or anxious about its occurrence, high arousal in response to anticipation to events such as meals, walks, and cleaning can also stimulate barking. Again, the underlying resultant basic motivations are often frustration or fear, with consequential sympathetic nervous system activation. Other previous studies have shown that barking initiated by one dog can be amplified, passed on, or increased by other dogs who then also start to bark by social facilitation or contagion [10]. Consider the dog that barks, then a nearby dog barks back, which therefore stimulates the dog who originally initiated the barking to bark again in response. In some animals, barking in and of itself can act as a stimulus for further barking [10]. These types of barking pollution could occur whether a person is actually present in the ward or not. Barking is a complex issue and creates a vicious cycle because barking is both a cause and a result of the stress in kennel environments.

Traditional recommendations to manage sound pollution in shelters have focused mainly on structural or environmental changes. Most of the environmental recommendations consisted of adding sound dampening material on the walls or ceiling, although few shelters are able to undergo major renovations to manage sound. Additionally, many of these materials unfortunately are sensitive to moisture and are not amenable to deep cleaning, resulting in soiling of material, or short lifespan, especially if any infectious disease outbreak occurs that would require sanitation of room surfaces. Managing social stimulation is another area that has been studied as a strategy to reduce barking in dogs. A study showed that limiting visitor access can decrease barking and increase sedentary or relaxed behaviors [27], suggesting lower overall noise levels and more positive welfare states. However, confinement and little human interaction can also create frustration and negative emotional states for dogs and that can also lead to unwanted behaviors such as barking [7]. It is important to consider that in most shelter environments, dogs must be able to be viewed by the public in order to be adopted and experience a shorter LOS.

Several animal welfare organizations have produced clinical recommendations to decrease barking in kenneled dogs. Many recommendations outline the use of classical counterconditioning in order to change the negative emotional state (fear or frustration) to a positive emotional state. This is achieved when people passing through the kennels toss food treats to the dogs, regardless of the behavior of the dog and regardless of whether or not they are barking. This allows dogs to anticipate positive interactions from the people that pass by and changes the underlying emotional state (conditioned emotional response—CER) [7]. Over time, this should change the dog’s emotional state to a more positive one, and consequently the motivation behind the barking (negative emotional state) when people are present or anticipated to be so is reduced. This exercise is often referred to as the QKE. While many facilities have put these recommendations into practice and found them to be beneficial, this concept has not been fully evaluated in peer-reviewed scientific literature, although Protopopova and Wynne (2015) [28] have investigated this concept using a strict application construct in a shelter. Before discussing the details of that important study, it is important for readers to understand classical, or Pavlovian, conditioning. This occurs when a neutral stimulus (e.g., bell), one that has no inherent meaning to the dog, is repeatedly paired with a stimulus that does have inherent meaning (unconditioned stimulus, e.g., food.) Over time, the neutral stimulus (bell) becomes a predictor for the unconditioned stimulus (food), and the dog now responds to the bell in the same manner as it does to food (e.g., drooling). This response, known as the unconditioned response when occurring naturally with food, now is considered the conditioned response, when it occurs in the presence of the bell only. This associative learning is a largely unconscious process that results in pairing emotional and visceral responses with something without meaning that does not require the individual to focus on the association and can occur during times of high arousal and stress. This construct can then be specifically targeted into a behavior modification technique by pairing a conditioned stimulus previously paired with a negative emotional response. In this case, people entering the ward are associated with fear, anxiety, or frustration, resulting in barking. This is paired with something inherently pleasant—food. Over time, repeated pairing changes the meaning, or emotional association, of the person to one that is more pleasant, such as anticipation of food, and therefore the resultant behavior is one that is more associated with food, such as attending to the person, often sitting, and now a more positive conditioned emotional response. The readership is encouraged to review this process in Modules 2 and 3 of Fear Free Shelters (Appendix A).

In the Protopopova and Wynne (2015) study [28], they used differential reinforcement of another behavior (DRO), also known as operant counterconditioning (OCC), as the reference standard and no intervention as the negative control. For DRO, an auditory tone was played and the dogs were given a reinforcer (food treat) when they did any behavior that did not fall into the “unwanted” category (response dependent pairing). For response independent pairing (classical counterconditioning), they rang the tone and then tossed treats regardless of the dogs’ behavior. They found that both interventions showed statistically significant improvement in the presence of unwanted behaviors and were not statistically different from each other. They described using the tone as the initial unconditioned stimulus to start each session to ensure the dogs understood the pairing. However, they recognized that this would not be realistic in a real-time shelter setting and suggested removing this in future studies [28].

This study aims to be the first step in providing scientific proof of concept that the clinical recommendations outlined above, using the simpler construct of the presence of a person as the conditioned stimulus, are effective. The study focused on trying to decrease barking in kenneled dogs through a simple behavior modification technique of classical counter conditioning, termed here as the “Quiet Kennel Exercise”. The QKE utilizes the clinical recommendation of classical counterconditioning as described above by having passers through of the kennel ward give food treats to kenneled dogs each time they walk through, regardless of whether the dog was barking or not, similar to the response-independent pairing previously described [28]. We hypothesize that the volume of barking will be decreased, along with the number of dogs barking, when a person passes through a ward, after classical counterconditioning (in the form of the QKE) is implemented for a minimum of two weeks. This approach will provide the pilot evidence needed to inform a larger study in this area and support the use of classical counterconditioning in kennel environments to improve the welfare of dogs being housed.

## 2. Materials and Methods

### 2.1. Kennel Environment

This study was performed at the North Carolina State University College of Veterinary Medicine Health and Wellness Center (HWC) between the dates of 21 May 2019 and 11 June 2019. This pilot study was performed in Ward C, which is one of the wards available for faculty, staff and students to board their personal pets during the work day.

**Figure 1:**
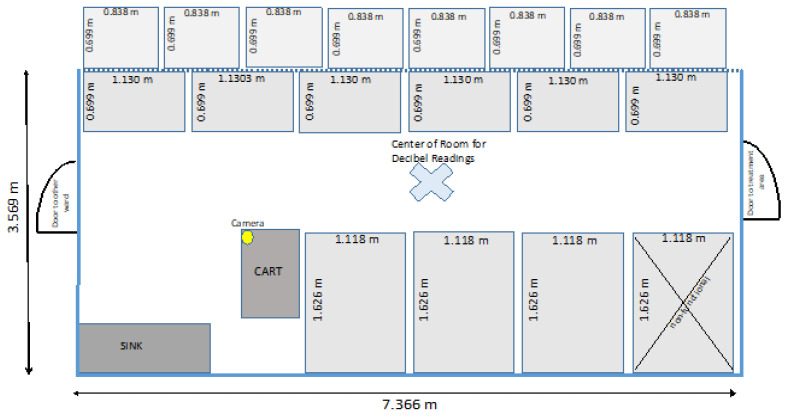
Footprint (layout) of study ward (Ward C).

The study ward (Ward C) consists of an enclosed room with one door at each end. One door enters into a hospital treatment room, and the other door opens into a separate boarding ward. The room itself is 7.366 m long, 2.642 m high, and 3.569 m wide. The walls of the room are made of painted cinder block, the floor is made of concrete, and the ceiling is made of standard dropped ceiling tiles, four air vents, and four fluorescent panel lights. The aisle way measures 1.219 m wide in the center of the room running the length of the room between two rows of kennels that are along the walls of the room. One corner of the room has a sink and cart. Ward C contains 18 kennels, 17 of which were functioning and available during the study period. The kennels are of three different sizes. Along the wall where the sink and cart are, there are four large kennels on one side of the room, which included the one kennel that was not functioning (three available large kennels). Along the opposite wall, there are six medium kennels at ground level with eight small kennels located above the medium kennels. The dimensions of the large kennels are 1.626 m long, 1.118 m wide, and 2.172 m high. The front and doors of the large kennels are made of chain link metal. The lower part of the side walls of the large kennels are made of painted cinder block measuring 1.219 m high, and the upper portion is made of chain link that measures 0.914 m high. The back wall consists of the painted cinder block of the wall itself, and with that, reaches the ceiling. The large kennels have concrete floors, and the top of the kennels are open and uncovered. The dimensions of the medium kennels are 1.130 m long, 0.699 m wide, and 0.826 m high. The dimensions of the small kennels are 0.838 m long, 0.699 m wide, and 0.597 m high. The small and medium kennels have vertical metal bars comprising the front door of the kennel, and the remainder of the kennel is made of fiberglass. For the study, each kennel had a one-quart metal bucket hooked to the outside of the kennel door to hold treats. The bucket was placed in the middle of each kennel door. The treat buckets were filled with 30–50 small food treats, ensuring that the owner’s requested preference was honored. The treat options offered for the study were Salmon, Chicken, or Bacon flavored treats (^a^ Zuke’s Mini Naturals^®^ Salmon Recipe (Durango, CO, USA) and ^b^ Pet Botanics MiniTraining Reward^TM^ Chicken Flavor and Bacon Flavor (Azusa, CA, USA), or owners were allowed to provide their own treats if preferred. Figure 1 shows the layout of the ward.

A webcam (^c^ Wyze Cam–Wyze Labs, Inc. (Seattle, WA, USA) was mounted on a tripod near the corner of the cart in order to record passersby of the ward and whether they participated in the QKE instructions. See Figure 1, where the yellow dot demarcates the placement of the webcam.

### 2.2. Dogs

Eleven different dogs were housed in Ward C throughout the duration of this study. Inclusion criteria were specified to dogs of any sex, intact or neutered, and any breed. Participating dogs had to be within six months to 15 years old and weigh between two and 120 pounds (0.9 to 54.5 kg) so that they could appropriately be housed in the size kennels available in the ward. Dogs had to be in good health and up to date on vaccines as required by hospital policy to board at the hospital. Additionally, dogs were required to be singly housed to avoid any potential aggression over the food since treats were being tossed into the kennels. Visual barriers, such as towels or blankets on the front of the kennel, were not to be used during the study period. Visual barriers could not be used because it was necessary to be able to toss the treats into the kennel and not have the barriers interfere with the results obtained. It was not a requirement for the same dogs to be present every day throughout the duration of the study. It also was not a requirement for the dogs to be housed in the same kennel each day they were present, as this pilot study was intended to mirror realistic settings for boarding kennels and shelters that have constantly changing populations. Because the dogs were managed by their owners during the study period, the researchers had no control over their daily schedules such as arrival time, departure time, meal times, or walks. Each participating dog’s owner signed a consent form prior to boarding their dog in Ward C during the study period.

### 2.3. Human and Animal Ethics

The study was approved by and performed in accordance with the Institutional Animal Care and Use Committee (IACUC-19-073-O) at North Carolina State University.

### 2.4. Noise Measurements

**Figure 2:**
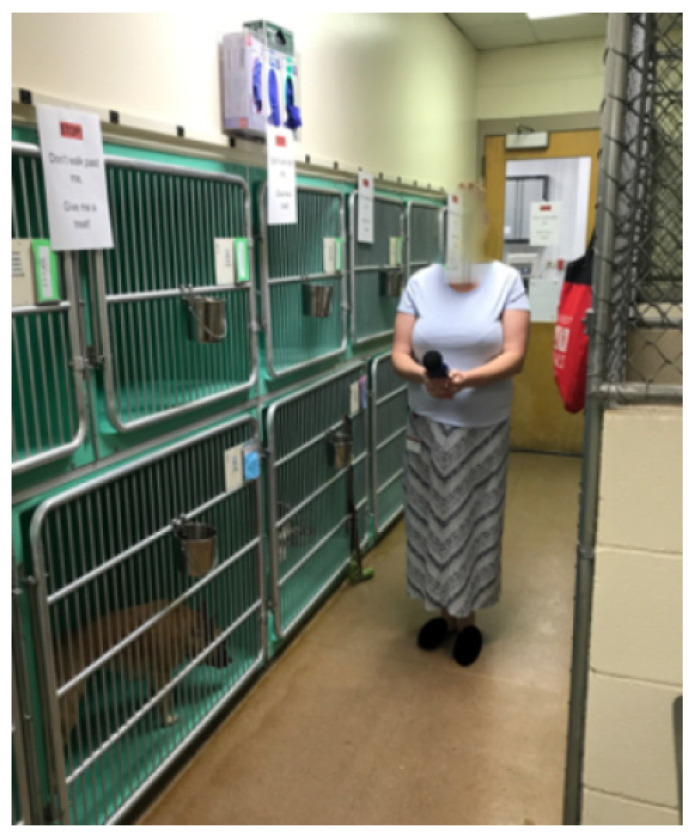
Position of Researcher for Sound Measurements. The position of the researcher corresponds to the “X” in Figure 1. Reproduced with permission from Stephany Spano (photographer) and Samantha Zurlinden (subject), created by Stephany Spano (July 2019).

The researchers used ear plugs (^d^ MACK’S Slim Fit^TM^ Soft Foam Earplugs NRR 29 dB mcKeon Products, Inc., Warren, MI, USA) for hearing protection, and data were collected three times daily (morning (AM), mid-day, and afternoon (PM)) for five days (Monday through Friday) for three weeks (data collected over 15 days total). The volume of barking was recorded using a handheld decibel reader (^e^ BAFX Sound Meter, Model: BAFX3608- USA (Muskego, WI, USA). It was set to measure a range of 30–130 decibels (dB) with fast time weighting. Fast time weighting picks up instantaneous real-time readings versus slow time weighting which records a reading that is the average decibel within one second. The frequency weighting was set to dBA, which is intended for general sound level measurements, rather than dBC, which is weighted to identify the low-frequency content of sounds. Each of the three data collection periods with the decibel reader were recorded for 30 s in the center of the ward (location demarcated with the large X in the middle aisle visualized on Figure 1 and is shown photographically in Figure 2, where the researcher is standing at X). The data from the decibel reader was then uploaded to SoundLab Advanced Sound Meter software ^f^ (BAFX Products^®^ USA (Muskego, WI, USA) after each reading, and each individual measurement was recorded to obtain the minimum, maximum, and average dB reading. During the 30 s of sampling, the number of dogs present in the ward as well as the number of dogs that barked during that period was recorded using a continuous sampling method.

### 2.5. Quiet Kennel Exercise (QKE)

During the initial five-day period, baseline data were collected as described above. During this time, people passing through the ward were asked to act as they normally would. After the initial baseline period, the QKE intervention began. The QKE intervention utilizes the concept of classical counterconditioning, and anyone passing through the kennel ward was asked to simply give each dog a treat from their respective treat bucket on their kennel, regardless of the dog’s behavior in the kennel. The person did not need to stop or say anything or wait for any specific behavior. Signage was placed on the doors to the kennel ward to request participation in the QKE. If the passerby was not willing or able to participate in the QKE, they were asked to remain neutral and completely ignore the dogs as they walked through. If unable to do this, they were instructed to use an alternate route to their destination rather than walk through Ward C. In addition, two researchers completed 10 walks through the ward (five walks each) each day of the intervention period. Of these 10 walks per day, the researchers participated in QKE seven times, while remaining Neutral during the other three. The order of the 10 walks for QKE versus Neutral was randomized using a random number generator [29]. The purpose of these 10 walks, with three of them being neutral, was to estimate compliance at a typical boarding or shelter environment where not all passersby would be willing or able to perform the QKE every time they go through a kennel.

To monitor the number of pass-throughs, defined as the number of times a person walked through the ward, and the compliance of people participating in the QKE while passing through Ward C during the intervention period, the previously described webcam was set to record those passing through the ward throughout each day and whether they performed QKE or Neutral behavior. The video was reviewed by researchers and the total number of pass-throughs were counted each day, as well as how many of the pass-throughs participated in the QKE, and how many remained neutral.

Decibel readings were collected three times a day using the same decibel reader with the same measurement setting used during baseline data collection for the QKE intervention period. During these decibel readings, the investigators were counted as Neutral since they did not interact with the dogs. The random number generator [29] was also used to assign decibel readings randomly to other investigators not involved with the QKE baseline data collection. This resulted in a more novel person collecting decibel readings once or twice per day during the QKE period. This was done with the intention to limit the dogs’ potential anticipation and habituation towards familiar individuals who may be associated with giving treats.

### 2.6. Statistical Analysis

The data were analyzed using JMP Pro statistical software (version 14.1.0, Cary, NC, USA). Summary and descriptive statistics were calculated, and data were inspected for outliers. The response variables that were analyzed include the mean, median, minimum, and maximum of the decibel measurements at each reading each day, the number of dogs barking, and the proportion of dogs barking. Because all data were collected on the same ward, the statistical analysis was largely limited to descriptive statistics and exploratory analyses. As part of the exploratory analysis, trend lines were fit through the baseline data and the QKE periods for the median, minimum, and maximum decibel recordings and the trends were compared. The fold reduction, which is the ratio of the difference in noise from baseline to the end of the intervention, of the QKE periods was also calculated.

## 3. Results

### 3.1. Study Participants

#### 3.1.1. Dog Participants

Eleven dogs participated over the course of the 15-day study period. Eleven dogs were present over the course of the baseline period, and eight dogs were present over the QKE study period. However, as expected, not all 11 dogs were present each day of the study. Some of the dogs were habitually day-boarded in this ward on a regular basis, while others were boarded there only occasionally. The number of dogs present for sound measurements during the study ranged from two to eight individuals. Demographic information about the canine participants and the number of days each individual dog was present for the baseline and intervention period varied and are outlined in Table 1. The background volume of sound in the ward when no dogs were barking ranged from 50–60 dB.

#### 3.1.2. Dog Participant Body Language

Subjectively, researchers noticed changes in dog body language after the QKE was implemented. During the baseline week when the QKE was not yet implemented, many of the dogs displayed body language that was indicative of fear and frustration. Several dogs were stiff and tense throughout their body, with their ears pinned back, tails tucked, and frequently barking and sometimes growling. There were noticeable body language changes indicative of an improved emotional state after the QKE was implemented. Dogs were observed wagging their tails and had noticeably more loose muscle tension through their body, with their ears forward, facial musculature softer, and appeared more attentive when a person entered the ward. Growling was no longer observed or noted in any study participant. Though body language was not a parameter that was measured in the study, several people who passed through the ward remarked on the improved body language. There are two videos in the supplementary data that show one dog’s body language during the baseline week before QKE intervention (Video S1), and one video recorded during QKE intervention in the same dog (Video S2).

### 3.2. Barking Data

#### 3.2.1. Volume (dB) of Barking

**Figure 3:**
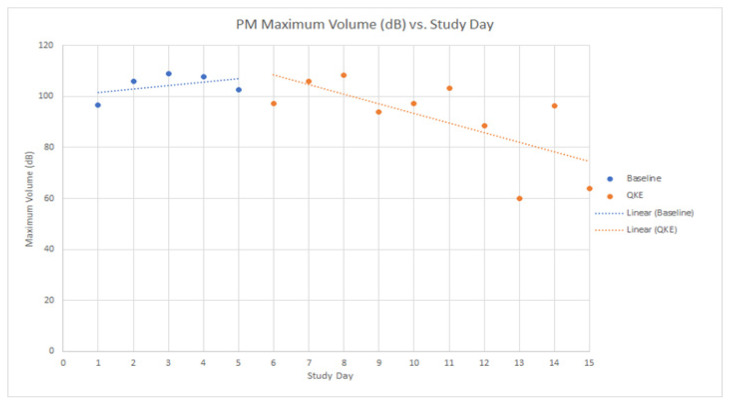
Maximum Volume (dB) vs. Study Day for PM (afternoon) sound measurements.

The maximum volume (dB) of barking decreased during the 10 study days of the QKE intervention with the largest reduction of volume in the PM readings (red line on Figure 3), which is interesting to compare to the PM readings during the five days of baseline data collection, which are shown in blue on Figure 3 below. For other times of day (AM, Mid-Day) and volume measurements (maximum, mean), trends were not apparent in baseline or intervention time periods in the descriptive data when reviewing the volume of barking alone.

Additionally, it was interesting to compare the average volume of sound (dB) and the number of dogs barking. For example, on study day two during baseline data collection, the average reading was 95.47 dB during the PM reading with three dogs barking. However, when three dogs were barking in the PM reading on study day 13 during the QKE period, the average dB reading was 69.47 dB. Though not statistically significant, there is a clear trend of decreasing volume despite the same number of dogs present.

#### 3.2.2. Duration and Volume of Barking—Baseline vs. QKE

**Figure 4:**
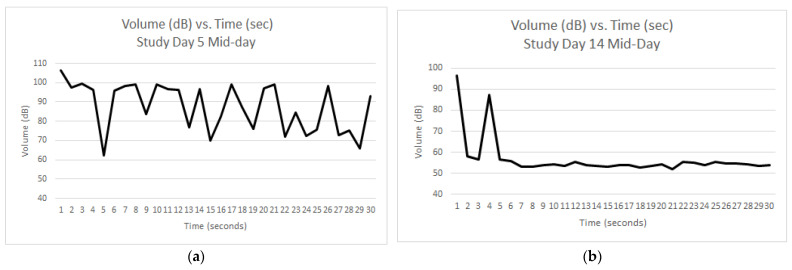
Volume in dB of barking over the 30 s time interval for two different study days. On both days, three dogs were present, and only one was barking during measurement; (**a**) represents the mid-day measurement on Day 5 (last day of baseline), whereas (**b**) represents the mid-day measurement on day 14, near the end of the QKE intervention implementation.

Graphs were generated for each reading with the decibel reader. During the baseline week of data collection, many of the graphs looked similar to Figure 4a, where the barking continued throughout most, if not the entirety, of the 30 s reading. At this particular reading, there were three dogs present, one of which was barking. In the graphs, each spike represents a discrete bark. During the QKE study period, the graphs showed that barking typically occurred during the initial three to five seconds of the 30-s reading period, and then decreased to background dB levels for the rest of the reading. Figure 4b is the mid-day reading on study day 14 during the QKE intervention where there were three dogs present, one of which barked during the 30 s measurement.

#### 3.2.3. Duration and Volume of Barking—Time of Day

**Figure 5:**
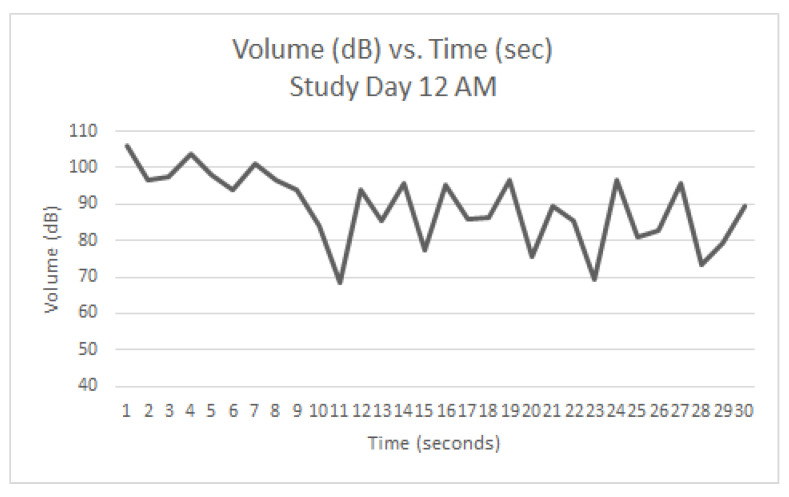
Study Day 12 (AM) during QKE (5 dogs present, 3 barking).

**Figure 6:**
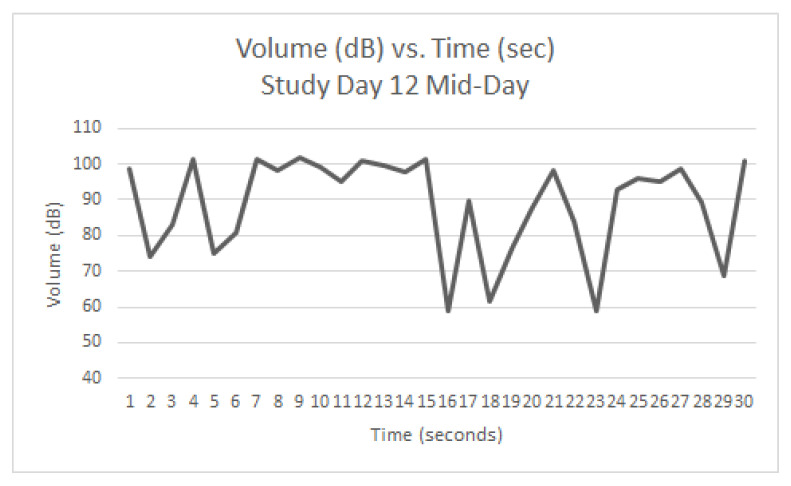
Study Day 12 (Mid-Day) during QKE (5 dogs present, 2 barking).

**Figure 7:**
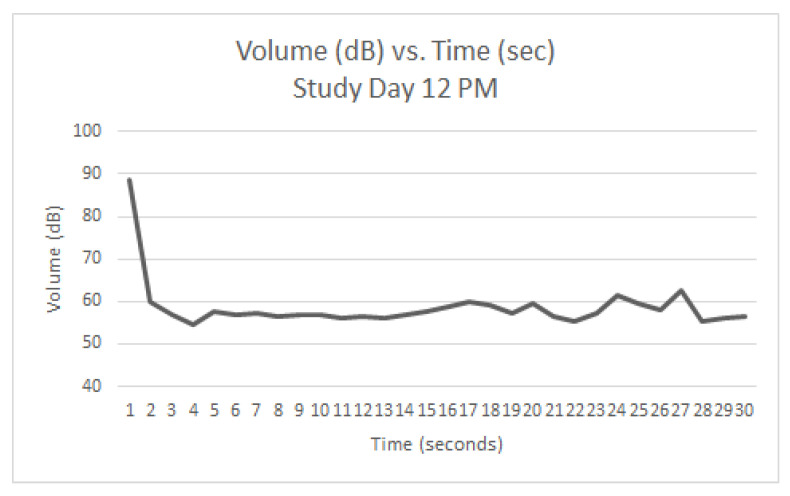
Study Day 12 (PM) during QKE (5 dogs present, 1 barking).

Graphs were generated from the readings taken on Study Day 12 (AM (Figure 5), Mid-Day (Figure 6), and PM (Figure 7). The same five dogs were present for the entirety of the day and all three readings. At the AM reading, three dogs were barking. At the Mid-Day reading, two dogs were barking. At the PM reading, one dog was barking. Each spike in the graph represents a discrete bark.

#### 3.2.4. Number of Pass-Throughs and Compliance of Participation in QKE by People Passing through Ward

During the QKE period, there were 27–51 pass-throughs in the ward per day, and the mean number of pass-throughs over the QKE study period was 38.9 pass-throughs per day. There is no clear trend of increased or decreased numbers of pass-throughs per day during the duration of the QKE period. Over the two weeks of the QKE intervention, the percentage of pass-throughs that participated in the QKE by giving the dogs treats (compliance) was 55.3%. During the first week of QKE intervention, the total compliance was 52.2%. During the second week, the total compliance was 58.5%. The total number of pass-throughs and the percentage of those who participated in the QKE for each day of the intervention are outlined in Table 2. The number of Pass-Throughs were not recorded during the baseline period (Study Days 1–5).

## 4. Discussion

The descriptive results revealed a reduction over time of maximum volume of barking in the PM measurements (see Figure 4, Figure 5, Figure 6 and Figure 7), as well as improvements of dogs’ body language and emotional states after the QKE was implemented. This cause of this is likely multifactorial. First, the ward and surrounding areas had less human activity and distractions in the afternoons as the work day ended, which contributed to less fear and frustration and hence less barking in the afternoon. This interpretation is supported by Hewison et al. (2014) [27], who found that preventing visitor access to kennels resulted in lower kennel noise levels and behaviors indicative of improved welfare. However, as previously discussed, this is in contradiction with the desire for dogs to be visible and accessible to visitors in order to facilitate adoption more easily. Therefore, the QKE could be an effective compromise to help achieve this outcome. It is important for readers to recognize that we are trying to address barking that is a consequence of people within or near the ward as the stimulus for barking, and that this exercise, in conjunction with other strategies to reduce negative emotional states and resultant barking, would be most effective to improve welfare overall. If time and resources were not a chronic limiting factor, determining the primary goal of barking for each dog and addressing the reason for the attempt at communication, either to conspecifics or to people [26], would create an ideal setting to meet the social needs and improve welfare for sheltered or kenneled dogs. Unfortunately, the reality of the vast majority of kenneled settings do not lend themselves to this level of evaluation and intervention. Second, this could be the result of normal circadian fluctuations in motivation to bark, similar to the diurnal pattern of barking identified by Sales et al. (1997) [3]. There, as here, barking was decreased in the late afternoon and evening. Third, it is possible that the dogs’ emotional states improved throughout the day as the QKE was implemented and the dogs were exposed to repeated sessions of classical counterconditioning (QKE) and pairing of people in the ward with receiving a food treat. It is important to acknowledge that since not all dogs were present each day of the study, it was possible that dogs present during intervention days still started each day in a similar emotional state as the dogs would during the baseline week. This helps explain how their emotional states would improve as each day progressed and why barking behavior, especially in the afternoon, was decreased. Finally, social facilitation is a common cause of barking, as previously discussed [3,10,25]. This could be a contributing factor in this study since there were multiple dogs in a contained ward. With the presence of fear, excitement, and frustration, especially in the morning, dogs would be more likely to bark, and one dog barking could lead to stimulation of social facilitation for more dogs to bark. As the QKE was implemented during the course of the day, social facilitation of barking would be decreased due to improved emotional states and acclimation.

The study conducted by Protopopova and Wynne (2015) [28] in a shelter setting utilized a bell as a conditioned stimulus to signify that food would be delivered, either using classical counter conditioning (response-independent treat delivery) or operant counterconditioning (differential reinforcement of other behavior—DRO or response-dependent treat delivery). Protopopova and Wynne (2015) [28] also were able to demonstrate that response-independent treat delivery was as effective as DRO, their reference standard. Additionally, they outlined several advantages that response-independent treat delivery has over response-dependent treat delivery (DRO) to improve behavior. These advantages include less time required to perform and less skill required by the person delivering treats. This is in line with our goal to help demonstrate that untrained passersby (staff, visitors, etc.) can become the conditioned stimulus and begin to predict a pleasant experience and therefore reduce unwanted kennel behavior, specifically barking. However, in the QKE experiment, no bell was used, and the presence of the person initially was the unconditioned stimulus and became the conditioned stimulus through repetition and pairing. This is consistent with one of the future study aims from that study [28], where they describe the presence of visitors or passersby being substituted for the bell.

Another interesting finding in our descriptive results was that the number of dogs barking did not necessarily increase the overall volume recorded by the decibel reader. For example, on Study Day two of the baseline period, the average reading was 95.47 dB during the PM reading with three dogs barking. However, when three dogs were barking during the PM reading on Study Day eight of the QKE period, the average dB reading was 69.47 dB. This is important since decibels are measured on a logarithmic scale [2,30]. For each 10 dB change in volume, there is actually a 10-fold change in sound intensity. So, going from 95.47 dB to 69.47 dB in volume is close to a 1000-fold decrease in sound intensity between these two readings. This decrease in sound volume and intensity would certainly help to improve welfare of all within hearing range and could be attributed to an improved emotional state due to the Quiet Kennel Exercise. Details are described in the results section and in Figure 4, Figure 5, Figure 6 and Figure 7.

Since the same dogs were not present each day, it was not feasible to quantitatively track individual dogs’ progression throughout the study period. However, study day 12 during the QKE intervention provided useful descriptive data as five dogs present that day were present for all three readings. Figure 5, Figure 6 and Figure 7, show the volume in decibels of the ward over the course of the three readings. The AM (Figure 5) and Mid-Day (Figure 6) readings had several discrete barks throughout the 30-s measurement. The AM reading had three out of five dogs barking and the Mid-Day reading had two out of five dogs barking. The PM reading (Figure 7) had only one out of five dogs barking, and it was only for the first few seconds of the reading. It is suspected that this improvement was due to improved emotional states of the dogs as the day progressed due to the QKE intervention and less opportunity for social facilitation.

During the 10 study days of QKE intervention, people walking through the kennel ward participated in the QKE intervention by tossing the dogs (termed compliance) 55% of the time. This means that 45% of the time people walking through did not toss treats to the dogs and remained neutral. This is in agreement with the author’s (SLB) clinical experiences of compliance that would be seen in a shelter or other kennel environment, where not every person walking by will be willing or able to toss treats to the dogs. As mentioned in the materials and methods, researchers participated in ten total walkthroughs per day, seven of which participated in QKE, and three of which were neutral, so the researchers had 70% compliance. However, the three times throughout the day that data were collected with the decibel reader were not counted as part of those ten walkthroughs. When compliance for the researchers was calculated, this would lower the researcher compliance to about 54%, which more closely matches the overall compliance we found in our study. While it would be ideal to have 100% compliance, as it may lead to more effective and efficient decreases in volume and improved emotional states, this is not practical or realistic in shelter settings. It was a goal of this pilot study to show that the QKE can be effective in real-life situations. In the Protopopova and Wynne (2015) [28] study they reported that for one dog who underwent response-independent training and extinction trials, it took four exposures without response-independent training to return to baseline levels of undesirable behavior [28]. This supports the premise that behavior can be altered and improved by intermittent or variable reinforcement schedules as is often the reality of implementation in shelter environments, adding further support that the QKE could be a practical solution for some forms of barking in the presence of people.

Interestingly, compliance was found to increase during the duration of the QKE period. During the first week, compliance was 52.2%, and during the second week of QKE intervention compliance was 58.5%. Though this is not statistically significant, it is hypothesized that those walking through the kennel ward were more willing to give treats as part of the QKE once they were able to see the impact of their participation in the exercise by a difference in the volume of barking.

There are several welfare implications for animals (dogs and other species) and people (staff, volunteers, visitors, and potential adopters) in the vicinity of high volumes of sound in kennel environments [2], in which the QKE could be a beneficial tool.

The volume of sound created from barking is a notable negative welfare implication for the dogs themselves as previously described, and has negative consequences on their sense of hearing. Dogs and cats have more sensitive senses of hearing than people, so it can be assumed that noise levels that negatively impact people also negatively impact animals [4,20]. Scheifele et al. (2012) [4], as previously described, found hearing loss that was of greater magnitude than the current level considered of concern for people for all 14 dogs that were kenneled for 6 months with continuous kennel noise levels greater than 100 dB. The Occupational Safety and Health Administration (OSHA) [30] guidelines would have required hearing protection for the people exposed to that level of noise, so the findings of this study are very concerning for the dogs exposed to high volumes of sound such as barking in kennel environments. The authors concluded that noise abatement strategies are necessary for kenneled dogs, especially those in long-term housing [4]. This concern is particularly relevant in shelter dogs, many of which have a long LOS. Ideally, recommendations to ensure physical and behavioral health and well-being for long-term care should be implemented as soon as possible, regardless of LOS expectations, but this should be especially prioritized whenever a stay is anticipated to exceed 1 or 2 weeks [20]. This is where the QKE can be a practical, efficient, and simple intervention to implement for every dog upon intake, in an effort to help reduce noise pollution from barking.

A frequent question that is posed of classical counterconditioning exercises such as the QKE is whether giving the dogs food treats while they are performing fear-related behaviors or other unwanted behavior such as barking, jumping up, or any number of other undesired behaviors, is actually reinforcing the fear, barking, or other unwanted behavior. To answer this, it is necessary to discuss the difference between classical conditioning and operant conditioning. If operant conditioning was used in this study, the dogs would only have been given treats when they were quiet. It is important to note that this can be an effective training strategy, as demonstrated by [28]. While both response independent (classical counterconditioning) and operant counterconditioning (DRO) schedules were effective for some dogs, the reward-dependent (DRO) training was likely only successful for dogs with a positive emotional state to begin with [28]. When dogs are in a negative emotional state (fear, anxiety, or frustration) and highly aroused, learning through operant conditioning is difficult and likely not achievable because the dogs are unable to focus on the trainer and learn the task (Yerkes–Dodson Law [31]. Additionally, inability to earn the reward during the training session can actually increase frustration and the negative behaviors motivated by it, further compounding the problem. But, once the dogs’ emotional states are normalized or moved into a more positive state through classical counterconditioning such as the QKE first, then operant conditioning could subsequently be utilized more efficiently and effectively to increase desirable kennel behavior, such as lying down or sitting when people pass by. This further supports the practicality of the QKE to improve dogs’ emotional states, and hence reduce barking behavior. As described in Protopopova and Wynne (2015) [28], operant conditioning in a shelter environment may not be practical due to the need for additional personnel training, precision in training technique, and the ubiquitous limitations of staffing hours, resources, and time in shelters.

An additional question that is often queried is whether the anticipation of food coming from the passerby through the ward will in and of itself increase frustration and consequent barking. While there is some possibility that some dogs that become frustrated very easily might have increased frustration as a result of food anticipation while awaiting the passerby to deliver the treat, this exercise when performed as described, should reduce frustration overall in a kenneled setting. Most frustration in a kennel setting results from anticipation of social interaction that is thwarted. With this exercise, each dog gets a predictable positive social response from the person as they pass by. The interaction happens to be in the form of delivering a food treat, which further strengthens the positive conditioned emotional response with the person present. It only takes a person a few seconds to go from kennel to kennel and deliver a food treat, so it is rare that the dog at the far end of a ward would begin to bark in frustration. On the contrary, clinical experience has shown that these dogs are more often than not going to stop barking in anticipation of the interaction and food treat. If the delay in treat delivery were much longer, this would be more likely to result in additional frustration of its own. This type of barking due to anticipatory frustration is more often observed during the sound of meal preparation in kennels and is common to many other species housed in groups, such as horse barns.

In addition to being a simple and practical behavior modification method, the QKE can also be inexpensive and therefore more easily implemented for shelters with limited resources. After the initial investment of treat buckets for kennels, at a cost of ~USD 8–10 per bucket, using high quality premium brand dog treats, the average cost per day in this study was USD 0.40/dog/day. The use of premium dog treats is not necessarily a requirement for the QKE. The cost could be reduced further by using palatable, but less expensive treats, or even the dogs’ own kibble.

While the descriptive data show several trends that suggest improvements in the dogs’ emotional states and decreases in the level of barking, the statistical analysis did not support the hypothesis for this pilot study. However, the hypothesis was necessarily negated due to the fact that this pilot study had several limitations. The sample size was small, and after additional statistical review, it was determined that the more appropriate experimental unit was the kennel ward rather than individual dog, further reducing the ability to apply meaningful statistical analysis. Additionally, there was variability in how many dogs were present each day, and which individuals were present. There was also variability in how often the QKE was performed by people walking through the ward. These variabilities were an intentional component of the study design in order to mimic a constantly changing shelter population and standard housing practices. The small number of dogs (11 dogs) was somewhat expected due to the number of kennels in the ward (17 kennels) that were available for study. However, researchers were also limited by the study being conducted in the summertime when there are far fewer dogs being boarded in the wards at the veterinary school. Along those lines, there were fewer student or, faculty staff passing through the ward at that time to participate in the QKE. The high variability of people passing through the kennel ward and whether they participated in the QKE or not was also intentional, as this is the reality in shelter and kennel environments. The data collected from this pilot study will help direct further research.

A longer study period will also be beneficial in future studies, with at least one week of baseline data collection and four weeks of QKE implementation. The data collected here were used to calculate preliminary power studies, which predict that six wards will be sufficient to achieve 90% power to detect changes from 120 dB to 85 dB, over 2–4 weeks with anticipated standard deviation of 20 dB. Four wards are calculated to be enough to detect a change from 120 to 70 dB, again with SD of 20 dB. Additionally, to avoid any possible habituation towards researchers taking decibel readings, it would be valuable to adjust study methods and design to include a decibel reader in the ward rather than a person actively entering the ward to take measurements.

This pilot study also helped to identify other parameters to be included in future studies. It would be beneficial to record demographic information about the people who pass through the ward, regardless of if they participate or not. Data could be collected regarding sex, physical features, or their role in the kennel/shelter to determine if this has any implication on the effectiveness of the QKE intervention. Additionally, future studies should collect data on the number of pass-throughs during both the baseline period and during the QKE period. In this pilot study, these data were only collected during the QKE intervention (Study Days 6–15). Collecting data on pass-throughs were not considered in the original study design, and it was amended to the protocol in time to be implemented during the QKE intervention only. Collecting data for the entire study period would provide valuable insight into whether the traffic through the kennels varies between the baseline and QKE intervention. It is possible that people may avoid the kennel so that they do not need to be concerned with participating or not. On the other hand, they may be encouraged to walk through more frequently as they realize that the QKE intervention is improving the barking behavior. The volume in dB could also be measured outside of the dog ward to determine the impact of noise pollution from barking on other species in a shelter environment. Potential areas to measure include the staff break room, small mammal housing, and cat housing areas of the shelter since loud volumes of sound can have negative implications to the individuals in these areas, in addition to those while in the ward. It also would be interesting to collect data on the incidence of infectious disease in dogs and cats in shelters during baseline collection and compare it to the same measures reported during the QKE intervention. As previously described, the volume of sound can increase stress levels in animals, which could lead to higher rates of infectious disease.

## 5. Conclusions

The QKE is a simple, practical, and inexpensive intervention for kenneled dogs that may have a positive impact on animal and human welfare by reducing barking in a short amount of time despite limited staff or resources. The QKE can benefit the dogs themselves, as well as other animals in vicinity of the barking. The concept of utilizing classical counterconditioning to change underlying emotional states that lead to barking is not a new concept and can be clinically very useful. It is challenging to provide proof of concept, as demonstrated by this pilot study. It is also important to educate the people involved in animal care and visitors that classical counterconditioning is effective. A brief explanation of the difference between classical and operant counterconditioning can be very powerful. This study is anticipated to be repeated on a larger scale with changes to the study design based on the results of this pilot study. The continuation of this research has been impacted and delayed by the COVID-19 pandemic.

## Figures and Tables

**Table 1:** Demographic data for canine participants.

Dog	Age (Years)	Sex ^1^	Body Weight (kgs)	Breed	Total Number of Days Present
A	3	FS	9	Terrier Mix	4 Baseline 0 QKE
B	8	FS	10	Mixed Breed	2 Baseline 6 QKE
C	2.5	MC	4	Papillon	3 Baseline 6 QKE
D	3.5	MC	13.5	Parson Russell Terrier Mix	4 Baseline 10 QKE
E	2	MI	7.25	Terrier Mix	4 Baseline 9 QKE
F	3	MC	17.25	Chihuahua	2 Baseline 0 QKE
G	10	FS	20.5	American Staffordshire Terrier	1 Baseline 0 QKE
H	1.5	FS	32	German Shepherd	4 Baseline 8 QKE
I	2	FS	22.75	Golden Retriever	3 Baseline 3 QKE
J	2	FS	10	Rat Terrier	1 Baseline 3 QKE
K	1.5	MC	41	Otterhound	1 Baseline 4 QKE

^1^ (F—female, M—male, I—intact, C—castrated, S—spayed).

**Table 2:** Total number of pass-throughs during QKE intervention, and total number and percentages of pass-throughs that participated in QKE.

Study Day	Total Pass-Throughs	Pass-Throughs that Participated in QKE Total # (%)
6	42	22 (52.4)
7	49	23 (46.9)
8	41	22 (53.7)
9	34	21 (61.8)
10	35	17 (48.6)
11	51	27 (52.9)
12	38	23 (60.5)
13	40	18 (45)
14	27	19 (70.4)
15	32	23 (71.9)

## Data Availability

Data has not been archived in a public dataset. It is available upon request of the corresponding author.

## Supplementary Materials

The following supporting information can be downloaded at: https://www.mdpi.com/article/10.3390/ani12020171/s1, Video S1: Baseline Video, Video S2: QKE Video.

## Appendix A

Fear Free Shelter Program: https://fearfreeshelters.com/program/ (accessed on 15 November 2021).

Tools included in Materials and Methods

a Zuke’s Mini Naturals^®^ Salmon Recipe (Durango, CO, USA) https://www.zukes.com/dog-treats-and-chews/training/mini-naturals-salmon-recipe (accessed on 15 November 2021).
b Pet Botanics MiniTraining Reward^TM^ Chicken Flavor and Bacon Flavor (Cardinal Pet Care, Azusa, CA, USA) https://www.petbotanics.com/training-reward-treats (accessed on 15 November 2021).
c https://wyze.com/shop-wyze#homeMonitoring (Seattle, WA, USA) (accessed on 15 November 2021).
d MACK’S Slim Fit^TM^ Soft Foam Earplugs NRR 29 dB mcKeon Products, Inc. Item #9150; 25460 Guenther, Warren, MI 48091 (586) 427-7560 www.MacksEarplugs.com (accessed on 15 November 2021).
e BAFX Sound Meter (Model: BAFX3608-USA) (Muskego, WI, USA) https://bafxpro.com/products/bafx-products-decibel-meter-sound-level-reader-w-battery-advanced-sound-meter (accessed on 15 November 2021).
f SoundLab Advanced Sound Meter software (BAFX Products^®^ USA; Muskego, WI, USA) https://bafxpro.com/products/bafx-products-decibel-meter-sound-level-reader-w-battery- (accessed on 15 November 2021).
